# Supplementary figures and images for: Microbiomes of Blood-Feeding Arthropods: Genes Coding for Essential Nutrients and Relation to Vector Fitness and Pathogenic Infections. A Review
Source: Microorganisms. 2021 Nov 25;9(12):2433. doi: 10.3390/microorganisms9122433 (PMC8704530; doi:10.3390/microorganisms9122433)

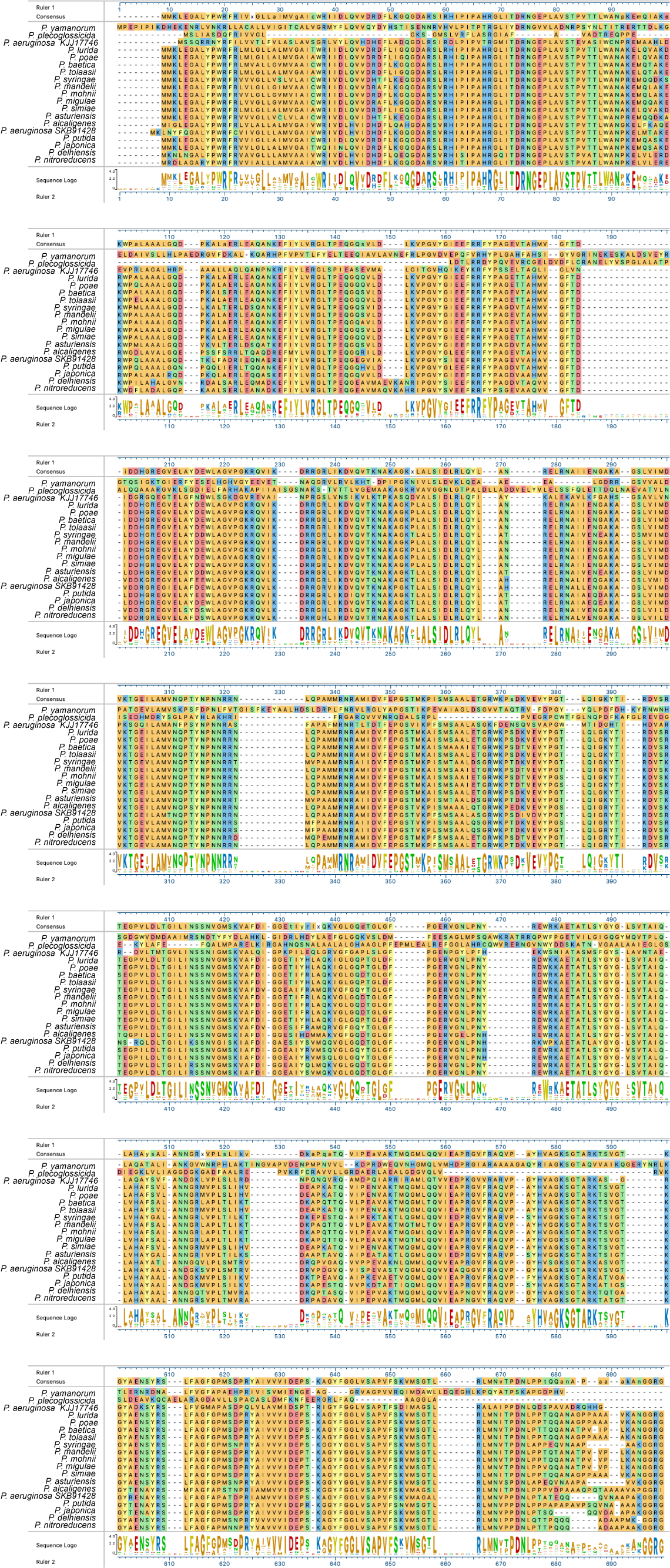

Supplement: Supplementary file 1 [file microorganisms-09-02433-s001.zip › microorganisms-1377975 revised version supplymentary/Supplementary Material/Supplemental Figure S1 Pseudomonas Clustal Omega amino acid alignment MEGA.png]
